# Childhood adversity and healthy ageing: a study of the Chinese older population

**DOI:** 10.1007/s10433-021-00608-8

**Published:** 2021-03-09

**Authors:** Bo Hu

**Affiliations:** Care Policy and Evaluation Centre (CPEC), Department of Health Policy, London School of Economics and Political Science, 8.01 Pankhurst House, Clement’s Inn, London, WC2A 2AZ United Kingdom

**Keywords:** Healthy ageing, Childhood adversities, Life course perspective, Gender equality, K-means clustering

## Abstract

This study examines the relationship between childhood adversities and healthy ageing in the Chinese older population. The data come from the China Health and Retirement Longitudinal Survey (CHARLS), a national survey that collected life history and ageing-related information on 9248 older people aged over 60 in 2014 and 2015. The analysis of healthy ageing focuses on seven indicators: IADL limitations, ADL limitations, cognitive functioning, depressive symptoms, life satisfaction, self-reported health, and chronic illness. Using k-means clustering, an unsupervised learning technique, we identified four qualitatively different groups according to their achievement in healthy ageing. We studied 17 types of childhood adversities and found that experiencing multiple childhood adversities is associated with a lower probability of achieving healthy ageing. This relationship is moderated by age and gender. Women are more vulnerable than men to the negative impacts of childhood adversities. The threat of childhood adversities to healthy ageing is greatly attenuated among people aged over 80. We argue that the policy response to healthy ageing should not be confined to those services and programmes that directly target older people. Instead, healthy ageing can be better addressed by concerted efforts in different areas of social policy.

## Introduction

Healthy ageing is the cornerstone of human development in later life. It refers to the process of developing and maintaining the intrinsic capacities that enable well-being in older age (World Health Organisation 2015). An emphasis on the intrinsic capacities in the conceptualisation of healthy ageing points to the multi-dimensionality of health. Key domains of older-age health include functional capability, cognitive functioning, physiological health, mental health, social well-being, general health status, and healthy behaviours (Lu et al. 2019).

A massive body of literature has examined the contemporary predictors of healthy ageing (Caballero et al. 2017; Feng et al. 2015; Hu and Wang 2019; Mejía et al. 2017; Sowa et al. 2016). There is a wide consensus that older people’s health is strongly associated with their socio-demographic (e.g. age, gender, and social capital) and socio-economic characteristics (e.g. education, income, and wealth). More recently, life course theories have maintained that ageing is a lifelong process that starts at birth and that exposure to events or experiences in early life has profound health consequences at later stages of the lifecycle (Alwin 2012; Elder et al. 2003). Following the life course perspective of human ageing, there is a heightened interest in academic studies in the relationship between childhood experience and health in later life. Childhood is considered a critical or sensitive period in the course of human development. With intensive biological programming taking place in this period, toxically stressful events or experiences may have negative health consequences that are difficult to alter or impossible to reverse in adulthood (Hu 2021; Jacob et al. 2015).

Adversities in childhood take effect on health inter-temporally through both direct and indirect pathways. The direct pathways involve the accumulation of toxic stress and are manifested at two different levels. At the biological level, exposure to adversities activates the stress-management system in the body. Frequent activation and deactivation of the system may affect the balance of the physiological state and the development of the body and brain, which is followed by a multitude of health problems such as cognitive impairment, lowered self-regulation, and vulnerability to physical and mental illness (McEwen and McEwen 2017). At the cognitive and psychological level, repeated exposure to adverse events or experiences compromises self-image and self-esteem (Pearlin 1999; Pearlin et al. 1981). Negative self-image and low self-esteem precede mental health disorders such as depression and anxiety (Patton 1991; Sowislo and Orth 2013).

The indirect pathways involve the proliferation of adversities in successive life stages. On the one hand, disadvantages in relation to cognitive development and impaired mental health caused by childhood adversities prevent children from fully benefiting from the education system, which in turn heightens the risks of unemployment or economic hardship in adulthood. Studies show that experiencing childhood adversities is associated with lower socio-economic conditions or social functioning in adulthood or midlife (Brimblecombe et al. 2018; Currie and Spatz Widom, 2010; Landes et al. 2014; Wolke et al. 2013). On the other hand, lower socio-economic status in midlife is associated with markers of early ageing (Foverskov et al. 2020), a higher mortality rate (Rehnberg and Fritzell, 2016), a higher probability of mobility limitations and depressed mood (Groffen et al. 2013), and an accelerated decline in cognitive performance (Ihle et al. 2018) in older age. In sum, socio-economic status and social capital in adulthood mediate the relationships between childhood adversities and older-age health.

The existing studies have focused on the health consequences of childhood adversities for older people as a whole, and there is scant evidence on how those consequences vary according to people’s demographic characteristics such as gender and age. Men and women differ significantly in terms of their biological response to stress, stress appraisal, and coping strategies (Matud, 2004; Ptacek et al. 1992; Rieker and Bird 2005). These differences often put females at a disadvantage in the face of highly stressful events or traumatic experiences. In addition, women in adulthood may face more gender-related adversities than men such as sex discrimination, domestic violence, or caregiving, which compromise their ability to cope with stress. Consequently, women may be more affected by childhood adversities than men.

Evidence abounds that older people are better at identifying the positive side of past events and moving away from negative memories or information than younger adults, not least because of the experience and wisdom accumulated throughout the life course (Blanchard-Fields 2007). Being mindful about the diminishing time in life, older people, especially those in very old age, shift their motivation from pursuing expansive goals to pursuing emotionally meaningful ones, such as deepening existing relationships, being grateful towards life and enjoying the present (Carstensen 2006; Ness et al. 2014). These age-graded changes in life perspectives and goals can protect people from the adverse impacts of childhood adversities.

In this paper, we study the relationships between childhood adversities and healthy ageing in the Chinese older population. China is facing unprecedented population ageing (Hu 2020). At present, there are 249 million older people aged over 60, approximately equivalent to the combined population of the UK, France, and Germany. The last three decades have seen the average life expectancy at birth increasing from 71 to 77 years (United Nations 2019). To make sure that longer lives go in tandem with healthier lives, there is an urgent need for studies in this country that concentrate on the determinants of healthy ageing, especially from a life course perspective. Based on the discussion above, we test the following three hypotheses in this study:

### Hypothesis 1

Childhood adversities are inversely associated with the achievement of healthy ageing.

### Hypothesis 2

Childhood adversities are more damaging to healthy ageing among women than men.

### Hypothesis 3

The negative impacts of childhood adversities on healthy ageing dissipate with age.

## Methods

### Data

The data came from the China Health and Retirement Longitudinal Study (CHARLS), a national survey that collected ageing and health-related information from a nationally representative sample of Chinese older people. The CHARLS followed the design of the Survey of Health, Ageing and Retirement in Europe (SHARE) and the Health and Retirement Study (HRS) in the USA. The baseline survey of the CHARLS took place in 2011, with two follow-up surveys conducted in 2013 and 2015, respectively, and a life course survey conducted in 2014.

The CHARLS recruited 8906 older people aged over 60 in the baseline survey, of whom 50.1% were female. The mean age of the baseline sample was 68.4 years old. For both follow-up surveys, a refreshed sample was added to ensure that the follow-up samples remained representative of the Chinese older population. The life course survey asked respondents a wide range of questions relating to their childhood experience. Our analysis focuses on the 9248 older people aged over 60 who participated in both the life course survey and the CHARLS 2015.

### Health indicators

We examined seven health indicators that relate to the four domains of healthy ageing: functional capability, mental health and well-being, cognitive functioning, and physical health. For functional capability, the CHARLS asked respondents to report their ability to perform six activities of daily living (ADLs) and six instrumental activities of daily living (IADLs). The six ADLs were dressing, bathing, eating, transfer, continence, and toileting (Katz et al. 1970). The six IADL were doing housework, cooking, shopping, making phone calls, taking medication, and managing money (Lawton and Brody 1969). For each ADL or IADL task, the functional capability was measured on a four-point scale, 1 = I have no difficulty in doing it, 2 = I have difficulty but still can do it, 3 = I need help, and 4 = I cannot do it. We added up the scores for the ADL and IADL tasks and created two variables that measure the ADL and IADL capabilities, respectively. A higher score indicates more severe functional limitations.

The CHARLS questionnaire includes a shortened 10-item version of the Centre for Epidemiologic Studies Depression Scale (CES-D) (Radloff, 1977). Respondents were asked to evaluate eight negative statements (e.g. I felt depressed) and two positive statements (e.g. I was happy) on a four-point scale: 1 = less than one day and 4 = five to seven days. By reverse-scoring the two positive statements and adding up the scores for the ten items, we created a variable measuring the severity of depressive symptoms. A higher score indicates more severe symptoms. Psychometric analyses conducted by Chen and Mui (2014) show that the 10-item CES-D scale is suitable to study the Chinese older population. The respondents were asked to evaluate their overall satisfaction with life on a five-point scale: completely satisfied, very satisfied, somewhat satisfied, not very satisfied, or not at all satisfied. We created a life satisfaction variable with five categories.

There are two modules in the CHARLS questionnaire that test older people’s cognitive functioning: mental intactness (numerical ability, time orientation, and picture drawing) and memory (self-rated memory and word recall). We analysed all of the questions in the module of mental intactness (14 items). Due to a substantial proportion of missing values in the word recall questions (proportion of missingness > 46%) in the module of memory, we only included self-rated memory (one item) in the main analysis. By adding up the number of incorrect answers to the 15 cognitive functioning questions, we created a continuous variable. The value of the variable ranges from 0 to 15, with a higher score indicating more severe cognitive impairment. Table 5 in the appendix provides a list of the 15 cognitive functioning questions we used to construct this variable.

We examined two variables relating to physical health. Respondents were asked to report whether they had any of the 14 chronic health conditions such as hypertension, diabetes, stroke, heart disease, asthma, etc. We added up the number of reported chronic illnesses. The questionnaire asked respondents to evaluate their overall health on a five-point scale: very good, good, fair, poor, and very poor. We created a self-reported health variable with five categories.

### Childhood adversity

We investigated 17 childhood adversities in the CHARLS questionnaire. They include (1) severe disability of a parent, (2) a bedridden parent, (3) a parent often feeling anxious, (4) a parent often feeling depressed, (5) a parent suffering from mental illness, (6) parental death, (7) divorce, (8) lack of affection, (9) parental neglect, (10) being physically abused by parents, (11) parental alcohol misuse, (12) parental drug misuse, (13) parental involvement in criminal activities, (14) frequent domestic violence, (15) being poorer than other families, (16) poor health in childhood, and (17) experiencing bullying victimisation in childhood. Following Schafer and Ferraro (2012) and Brimblecombe et al. (2018), we created a binary variable for each of these items (0 = no and 1 = yes), and then added up the total number of adversities for each respondent.

### Control variables

We controlled for socio-demographic and socio-economic factors that may confound or mediate the relationships between healthy ageing and childhood adversities. This enabled us to investigate whether childhood adversities affected healthy ageing independent of confounders or mediators. In terms of social-demographic factors, we controlled for age, gender, rural–urban residence, marital status, and household composition. Age is a continuous variable, while the other four are categorical variables. In terms of socio-economic factors, we controlled for older people’s level of education, housing tenure, and annual household income per capita. The education variable has three categories: no formal education, receiving primary education, and receiving secondary education and above. The housing tenure variable has two categories: owned housing and rented housing. We logarithmically transformed the income variable prior to the statistical analysis.

### Statistical analysis

The statistical analysis consisted of two steps. In the first step, we classified older people in the sample into qualitatively different groups according to their health profiles. We used the k-means clustering method, an unsupervised learning technique in the machine learning literature, to execute the classification (Hastie et al. 2009). The method partitions the sample by maximising the intra-group similarity and the intra-group dissimilarity. In our data, the similarity between two individuals $i$ and ${i}^{^{\prime}}$ in terms of the seven health indicators ($j=1\dots 7$) is measured by their squared Euclidean distance:

$$\mathop \sum \limits_{j = 1}^{7} \left( {x_{ij} - x_{{i^{\prime}j}} } \right)^{2}$$

A higher number of clusters reduces the intra-group dissimilarity but increases the model complexity. The optimal number of clusters strikes a balance between the two. This is achieved by following the ‘elbow rule’: we drew a scree plot of the within sum of squares and the number of groups, and the kink point in the curve was identified as the optimal solution (Makles 2012). The health indicators were normalised to a range between 0 and 1 beforehand to facilitate data visualisation. We used the ordered categorical variable derived from the clustering method to measure older people’s overall achievement in terms of healthy ageing.

In the second step, we investigated the associations between childhood adversities and the categorical healthy ageing variable. We first conducted Pearson’s χ^2^ test to examine whether each type of childhood adversity is significantly associated with healthy ageing. We then conducted an ordinal logit regression analysis to examine the relationships between the number of childhood adversities and the healthy ageing variable when the socio-demographic and socio-economic factors are controlled for. We included an interaction term between age and childhood adversities and an interaction term between gender and childhood adversities in the ordinal logit regression model to investigate the moderation effects of age and gender, respectively. Stata 15 was used for the data analysis.

In the base case analysis of the ordinal logit regression, we followed the conventional assumption of proportional odds and further assumed that there was no unobserved geographical heterogeneity in the data (syntax: *ologit*). In the sensitivity analysis, we relaxed the assumption of proportional odds by building a generalised ordinal logit model (syntax: *glogit2*) and accounted for the community-level unobserved heterogeneity by constructing a two-level ordinal logit regression model (syntax: *meologit*). Table 6 in the appendix shows the proportion of missing values for each variable. All of the variables where more than 5% of the values were missing were simultaneously imputed using the multiple imputation with chained equations (MICE) technique (syntax: *mi impute chained*). After imputation, 9139 out of the 9,248 observations in the sample were complete cases. The analysis results based on the complete cases were almost identical to those based on the full sample.

## Results

Table 1 summarises the sample characteristics. There were great variations in people’s childhood experiences. While 23% of the sample had not experienced any of the 17 childhood adversities under investigation, 14% had experienced three types of childhood adversities, and 16% reported more than four types of adversities. The average number of childhood adversities in the sample is 1.8. Among the 9248 older people in the sample, 62% (*n* = 5723) were in the age group 60–69, 29% (*n* = 2702) were in the age group 70–79, and 9% (*n* = 823) were aged over 80 in 2015. Seventy-nine per cent of the older people were married, and 89% were living with other people in the same household. Seventy-three per cent were living in rural China, and 30% had never received any formal education. The proportions of older people who were single or living alone were higher among those in the higher age groups. Older people in the higher age groups had a lower socio-economic status than those in the lower age groups.

**Table 1:** Sample characteristics

	Percentage (number of people) or means			
	Entire sample	Age: 60–69	Age: 70–79	Age: 80 +
*Health groups based on k-means clustering*				
Group 1	17 (1593)	18 (1052)	15 (414)	15 (126)
Group 2	39 (3580)	44 (2523)	35 (940)	14 (115)
Group 3	34 (3105)	31 (1768)	36 (986)	43 (352)
Group 4	10 (970)	7 (379)	13 (362)	28 (230)
*Number of childhood adversities*				
None	23 (2172)	23 (1,325)	23 (612)	29 (237)
1 adversity	26 (2419)	25 (1,455)	28 (747)	26 (211)
2 adversities	21 (1948)	21 (1,182)	22 (600)	20 (167)
3 adversities	14 (1262)	14 (805)	13 (345)	14 (114)
4 + adversities	16 (1447)	17 (956)	15 (398)	11 (94)
Age (years old)	68.7	64.0	73.8	83.9
*Gender*				
Male	49 (4533)	49 (2792)	51 (1378)	45 (368)
Female	51 (4715)	51 (2931)	49 (1324)	55 (455)
*Marital status*				
Single	21 (1984)	13 (763)	28 (762)	57 (465)
Married	79 (7264)	87 (4960)	72 (1940)	43 (358)
*Living arrangements*				
Living alone	11 (988)	7 (392)	14 (375)	27 (223)
Living with others	89 (8260)	93 (5331)	86 (2327)	73 (600)
*Rural–urban residence*				
Urban areas	27 (2494)	27 (1558)	27 (736)	24 (198)
Rural areas	73 (6754)	73 (4165)	73 (1966)	76 (625)
*Education*				
No formal education	30 (2801)	24 (1356)	35 (935)	61 (502)
Primary education	46 (4230)	49 (2814)	43 (1177)	30 (246)
Secondary education and above	24 (2217)	27 (1553)	22 (590)	9 (75)
*Housing tenure*				
Owned housing	84 (7767)	87 (4997)	80 (2149)	75 (618)
Rented housing	16 (1481)	13 (726)	20 (553)	25 (205)
Logarithm of income (Chinese Yuan)	8.3	8.3	8.3	8.0
Sample size	9248	5723	2702	823

**Fig. 1:**
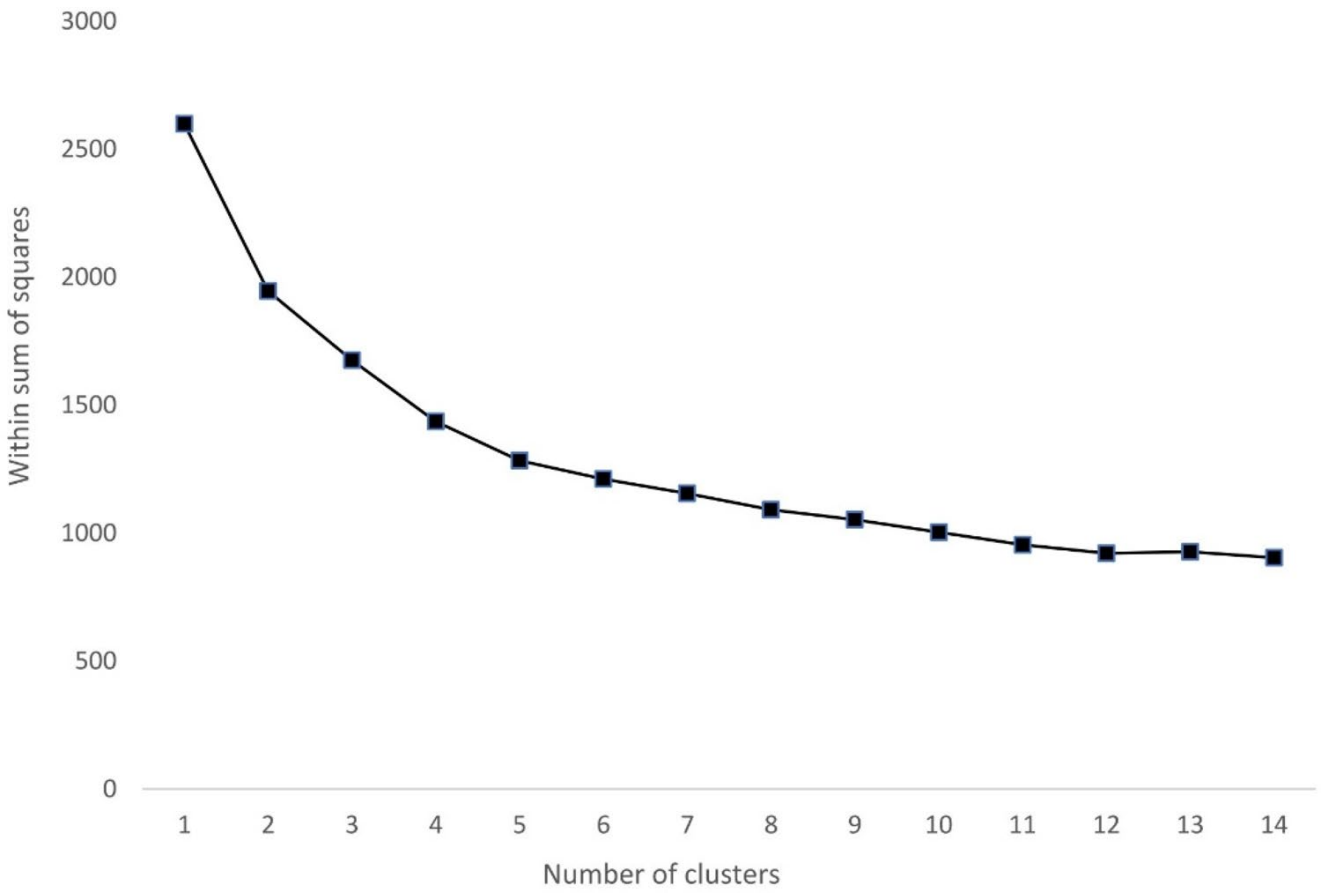
Within sum of squares for different number of clusters

**Fig. 2:**
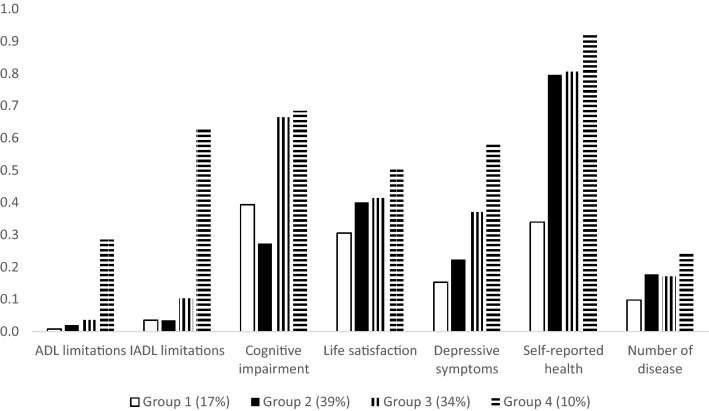
Health profiles of older people in the four clusters

The scree plot of the k-means clustering analysis showed that the within sum of squares (WSS) decreased quickly when the number of clusters was equal to or less than four, and the reduction became incremental thereafter (Fig. 1). Therefore, we selected the four-group model as the optimal solution. Figure 2 shows the health profiles of older people in the four clusters. A taller bar in the figure indicates poorer health in a particular dimension. Group 1 consists of the healthiest individuals in the sample, whereas group 4 represents the least healthy cluster. Overall, people in less healthy groups scored lower than those in healthier groups in every dimension of the measured health outcomes. About a quarter of the sample belonged to the two extreme groups, with 17% of people in the healthiest cluster and 10% in the least healthy cluster. The majority of older people belonged to groups 2 and 3. People in these two groups reported a similar level of ADL limitations, life satisfaction, self-reported health, and chronic diseases. However, people in group 3 had markedly worse IADL-based functional capability, lower cognitive performance, and more severe depressive symptoms than those in group 2.

A total of 12 out of the 17 childhood adversities were significantly associated with the categorical health variable (Table 2). All of the adversities relating to parental health and four of the parenting-related variables were significant risk factors associated with healthy ageing. Parental divorce, parental drug abuse, parental alcohol abuse, parental involvement in criminal activities, and frequently witnessing domestic violence were not significantly associated with healthy ageing. It is worth noting that the proportions of older people who reported parental divorce, parental drug misuse, and parental involvement in criminal activities were small. The non-significant associations may be attributable to lower statistical power.

**Table 2:** Univariate association between childhood adversity and healthy ageing

Childhood adversities	Percentage (number)	Pearson’s χ^2^ test for independence	*p* value
Severe disability of a parent	4.9 (453)	32.7	< 0.001
A bedridden parent	17.8 (1646)	46.9	< 0.001
A parent often feeling anxious	19.0 (1757)	40.3	< 0.001
A parent often feeling depressed	10.9 (1008)	115.5	< 0.001
Mental illness of a parent	3.7 (342)	25.0	< 0.001
Death of a parent	23.0 (2127)	9.6	< 0.05
Parental divorce	0.9 (83)	3.2	0.363
Physically abused by parents	5.5 (509)	10.9	< 0.05
Lack of affection from a parent	19.9 (1840)	14.4	< 0.01
Neglected by a parent	21.7 (2007)	21.5	< 0.001
Parental alcohol misuse	6.3 (583)	2.2	0.540
Parental drug misuse	0.5 (46)	1.9	0.584
Parental involvement in criminal activities	0.5 (46)	5.6	0.134
Often witnessing domestic violence	7.2 (666)	6.3	0.100
Poorer than other families	25.8 (2386)	113.8	< 0.001
Frequent bullying victimisation	4.1 (379)	12.8	< 0.01
Poor health in childhood	13.2 (1221)	28.0	< 0.001
Sample size	9248		

**Fig. 3:**
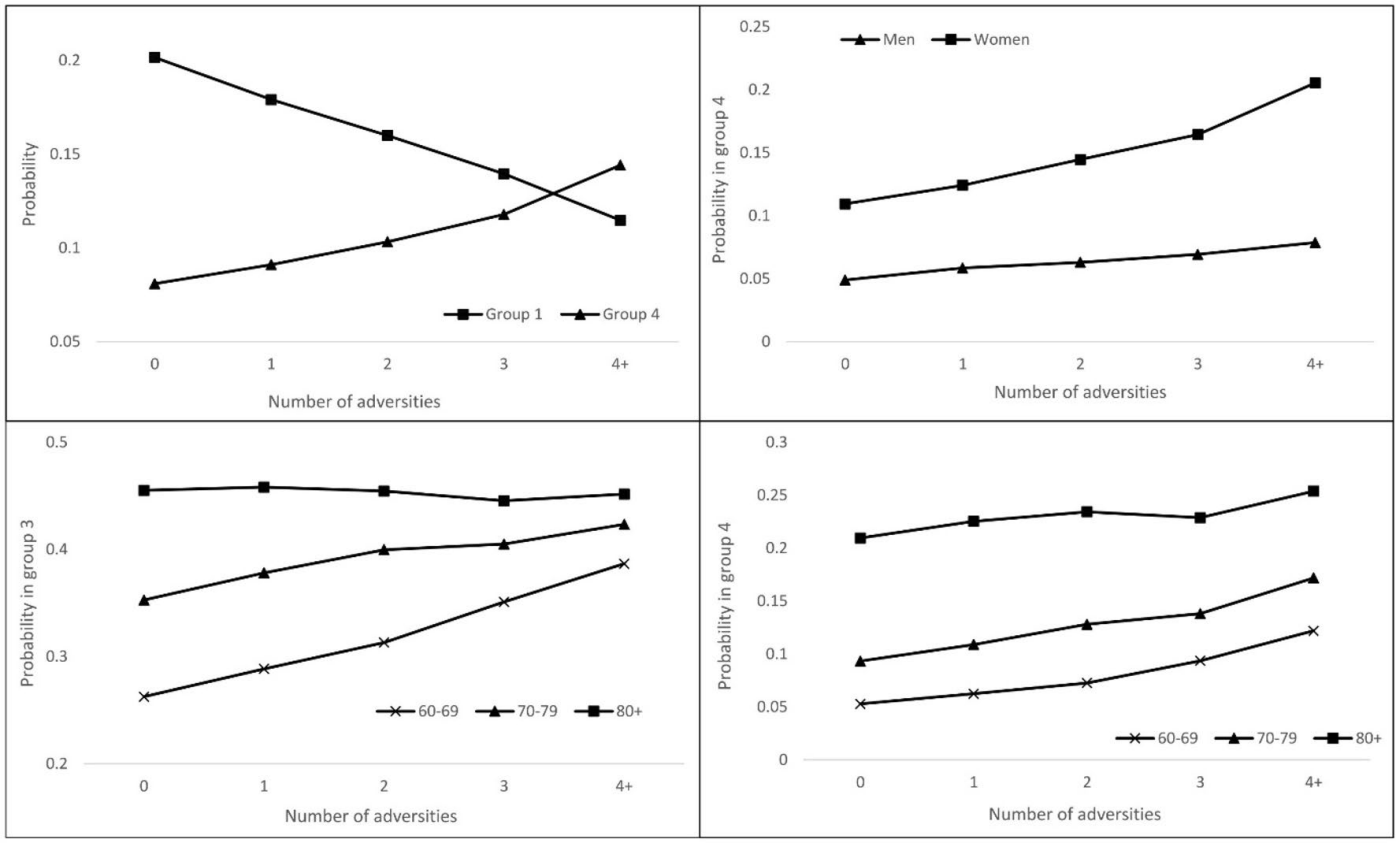
Childhood adversities and health in old age: the moderating effect of age and gender

The ordered logit regression shows that the number of childhood adversities was significantly associated with the health variable (Table 3). Experiencing multiple adversities in childhood was associated with poorer health in later life (odds ratio = 1.46, p value < 0.01). Among people who did not experience any childhood adversities, the probability of being in the healthiest group (i.e. group 1) was 0.20 and the probability of being in the least healthy group (i.e. group 4) was 0.08. For people who experienced more than 4 adversities, the probability of being in the healthiest group was 0.11, and the probability of being in the least healthy group was 0.14 (upper left panel, Fig. 3).

**Table 3:** Association between the number of adversities and healthy ageing: ordinal logistic regression

Independent variables	Odds ratio	95% confidence interval
Number of childhood adversities	1.46**	1.09–1.75
Age	1.05***	1.04–1.06
Female (ref. male)	1.43***	1.27–1.61
Adversity × Age	0.996*	0.993–0.999
Adversity × Female	1.10***	1.05–1.15
Married (ref. single)	0.80***	0.71–0.91
Living with others (ref. living alone)	1.05	0.91–1.23
Rural areas (ref. urban areas)	1.45***	1.32–1.59
Primary education (ref. no education)	0.46***	0.41–0.51
Secondary or above (ref. no edu.)	0.29***	0.26–0.33
Rented housing (ref. owned housing)	1.14*	1.02–1.26
Logarithm of income	0.94***	0.92–0.95
Sample size	9248	

*p < 0.05, **p < 0.01, and ***p < 0.001; ref.: reference category

Females were more likely than males to have poorer health (odds ratio = 1.43, *p* value < 0.001). Furthermore, the odds ratio of the interaction term between childhood adversity and gender was significantly larger than 1, indicating that the negative impacts of childhood adversity on healthy ageing were stronger among females than males. The moderating effect of gender is illustrated in the upper right panel of Fig. 3. As the number of childhood adversities increased from zero to four or above, the probability of being in the least healthy group increased from 0.05 to 0.08 among men, whereas the probability of being in this group increased from 0.11 to 0.21 among women.

People’s overall health declined with age (odds ratio = 1.05, *p* value < 0.001). Moreover, the odds ratio of the interaction term between childhood adversities and age was significantly less than 1, which indicated that the association between childhood adversities and health was weaker among older adults. For older people aged between 60 and 69, the probability of being in health group 3 increased from 0.26 to 0.39 (lower left panel, Fig. 3) and the probability of being in health group 4 rose from 0.05 to 0.12 (lower right panel, Fig. 3) as the number of adversities increased. This was in stark contrast to older people aged over 80, whose probability of being in groups 3 and 4 largely flatlined for different levels of childhood adversities.

Most of the control variables in the regression model were statistically significant (Table 3). Married people were more likely than single people to be in healthier groups. People living in rural areas on average had poorer health than those in urban areas. Socio-economic status was a significant predictor of healthy ageing. A higher level of education or income increased the probability of being in the healthier groups.

The regression results in the base case were highly robust to alternative modelling specifications (Table 4). When the assumption of proportional odds was relaxed, the main effect of childhood adversity in the generalised ordinal logit regression was slightly reduced but remained statistically significant (odds ratio = 1.39, *p* value < 0.05). Its interactions with age and gender were also statistically significant. The multilevel ordinal logit regression showed that community-level random effects were statistically significant (χ^2^ = 13.1, *p* value < 0.001), which means that there was great geographical heterogeneity in older people’s health status across the country. Nonetheless, the main effect of childhood adversities and the coefficients of the interaction terms remained statistically significant.

**Table 4:** Sensitivity analysis

	Odds ratio	95% confidence interval
**Generalised ordinal logit regression**		
*Group 2–4 versus group 1*		
Adversity	1.39*	1.08–1.79
Age	1.02***	1.01–1.03
Female	1.31***	1.13–1.51
Adversity score × Age	0.996**	0.992–0.999
Adversity score × Female	1.10***	1.05–1.15
*Group 3–4 versus group 1–2*		
Adversity	1.39*	1.08–1.79
Age	1.06***	1.05–1.07
Female	1.68***	1.47–1.93
Adversity score × Age	0.996**	0.992–0.999
Adversity score × Female	1.10***	1.05–1.15
Group 4 versus group 1–3		
Adversity	1.39*	1.08–1.79
Age	1.09***	1.08–1.1
Female	1.09	0.91–1.32
Adversity score × Age	0.996**	0.992–0.999
Adversity score × Female	1.10***	1.05–1.15
**Multilevel ordinal logit regression**		
Adversity	1.35*	1.06–1.73
Age	1.05***	1.04–1.06
Female	1.45***	1.28–1.64
Adversity score × Age	0.996	0.993–1.000
Adversity score × Female	1.10***	1.05–1.15

All control variables are included in the models;

*p < 0.05, **p < 0.01, and ***p < 0.001

## Discussion

This study investigated the relationship between childhood adversities and healthy ageing in the Chinese older population. The analysis results confirmed the three hypotheses of the study. We found that older people with stressful or traumatic experiences in childhood are less likely than people without those experiences to achieve healthy ageing. Furthermore, the detrimental effects of childhood adversities on healthy ageing vary markedly according to age and gender. Females and people in the younger age groups are most vulnerable to their detrimental effects.

### Childhood adversities as a threat to healthy ageing

The international literature has examined how childhood adversities are associated with separate domains of the health of older people. Landes et al. (2014) reported that a lack of parenting and a lower social status in childhood predict more functional limitations or morbidities in older age. Traumatic events in childhood such as physical abuse, sexual abuse, parental death, and bullying victimisation have been found to be associated with older-age anxiety or depression disorder (Chou 2012; Draper et al. 2008; Lund et al. 2008; Yang et al. 2019). Verropoulou and Serafetinidou (2019) and Weinstein (2016) found that poorer health and a lower socio-economic status are associated with more severe symptoms of affective suffering and motivation and lower functional capability. There is also evidence that a higher number of childhood adversities decreases the probability of disease avoidance (Schafer and Ferraro, 2012) and accelerates the disablement process (Shrira and Litwin 2014).

While confirming the previous studies, our study contributes to the existing literature by showing that childhood adversities can result in global damage to people’s intrinsic capacity: Chinese older people experiencing childhood adversities are more likely to score significantly worse in all of the key health indicators. Moreover, there is a significant dose–response relationship between adverse experiences in childhood and healthy ageing. The number of childhood adversities and the probability of healthy ageing is inversely correlated. The cumulative effect of multiple adversities is significantly more damaging than any single adversity. This association remains strong after we control for people’s socio-demographic characteristics, socio-economic status, and community-level unobserved heterogeneity. The implication is that childhood adversities take effect on healthy ageing independent of contemporary factors, which lends support to the theory that there is more than one pathway through which the two life stages are linked together. The independent effects of childhood adversities also underscore the importance of policy interventions that directly address risk factors and socio-economic inequality in childhood. Interventions targeting risk factors in midlife or later-life are crucial but not enough.

Healthy ageing is a gendered issue. Gender-based health inequality in older age has been reported in studies in European countries and the USA. Although the level of inequality differs from one country to another, women on average display a worse health status than men (Schmitz and Lazarevič 2020). Our study confirms such a gender difference: Chinese older women are significantly less likely than men to achieve healthy ageing. Like in other countries, women in China have a higher life expectancy and a lower level of education than men, which contributes to the poorer health status of women (Zhong et al. 2017). But even after we control for those factors in the analyses, Chinese women’s disadvantage in healthy ageing can still be clearly identified.

Gender-based inequality is further reflected in the heightened vulnerability of Chinese older women to childhood adversities. While the health status of men is less sensitive to an increase in the number of stressful experiences, multiple stressors greatly suppress women’s prospects of achieving healthy ageing. Such a finding provides empirical support to the existing theories that women differ from men in terms of biological response, stress appraisal, and coping strategies. Research shows that females’ immune systems have a more robust response to stressors than men, but this response may also attack their own bodies and put them at greater risks of health disorders than men (Rieker and Bird, 2005). For similar stressors, women tend to appraise them as more stressful than men (Ptacek et al. 1992). In response to stressful events, women are more likely than men to have strong emotional responses such as blaming themselves or feeling worthless and unimportant (Matud 2004). As a result, women face more damaging health and life consequences than men in the wake of highly traumatic events (Barker et al. 2008). The evidence in our study confirms this disparity from the perspective of healthy ageing.

Most importantly, our study shows that gender-based inequality is not limited to a particular life stage but spans the entire life course. Similar to women in other countries, women in China are in a disadvantaged position in terms of educational opportunities, social roles, and social expectations (Hannum 2005; Shu 2004). On the one hand, these disadvantages deprive them of the necessary resources to cope with stressful memories and rebuild their self-esteem and self-image. On the other hand, lower self-esteem and less positive self-image following childhood adversities may increase women’s vulnerability to gender-based discrimination or violence in adulthood, which in return exacerbates the health consequences of childhood adversities. It is not a particular type of adversity but the accumulation of multiple adversities throughout the life course that takes its toll on healthy ageing.

The analysis results confirm our hypothesis that the association between childhood adversities and healthy ageing is attenuated as people reach an older age. More childhood adversities are associated with significant health inequalities among people in the younger age groups, but they make little difference for people aged over 80. The findings are consistent with the prediction of the socio-emotional selectivity theory (Carstensen 2006). Compared to their younger counterparts, people in very old age tend to focus more on goals that are emotionally meaningful for the present rather than dwelling on the past. Ageing is a multi-dimensional and multi-directional process. Older people may experience a decline in health, but this does not prevent the continued growth of their socio-emotional skills. Due to the time left to live and the time lived, not only are they more motivated in this regard, but they are also more capable of doing so. A relatively positive attitude towards life or a sense of appreciation of the chance to survive to very old age protects Chinese older people’s mental and physical health from being further damaged by stressful memories of the past.

It should be noted that the socio-emotional selectivity theory is not necessarily the only explanation of the weakened association in the oldest-old population. An alternative explanation is the selection effect in the ageing process. With the help of both biological and environmental factors, people experiencing multiple adversities in childhood but managed to live to very old age may have developed resilience to the adverse impacts of childhood adversities (Crimmins 2005). Surviving the selection, they displayed health traits similar to those experiencing fewer adversities. To what extent the diminished differentials are driven by socio-emotional selectivity or mortality selection is a topic worthy of further investigations.

### Strengths and limitations of the study

Drawing on data from a large and nationally representative sample, this study focuses on the global impacts of childhood adversities on healthy ageing. An investigation of the social determinants of healthy ageing hinges upon a reliable way of capturing the multi-dimensionality of healthy ageing. Using k-means clustering, an unsupervised learning technique, we divided the Chinese older population into qualitatively different groups according to their health profiles. The categorical variable derived from this method provides an alternative approach to the measurement of healthy ageing and health inequalities, which differs from the latent factor approach used in the literature (Beard et al. 2016).

Our study has shown that this clustering-based approach has several merits. First, it is a purely data-driven method and thus frees researchers from the task of defining healthy ageing by setting thresholds for health scores. Sometimes the thresholds are inevitably arbitrary. Second, models based on the k-means clustering method are falsifiable. Established criteria exist that help researchers select the optimal solution from competing models. Finally, different health outcomes usually have reciprocal relationships. The morbidity, functional limitations, and mental health of older people reinforce each other in the disablement process (Verbrugge and Jette 1994). As a result, these health outcomes cluster together, and health inequality in the older population emerges and amplifies. The k-means clustering method is well-positioned to recognise these reciprocal relationships and represent them in the form of qualitative differences between clusters. Although we used this method to examine the pattern of healthy ageing in the Chinese older population, we expect it to be applicable to similar studies in other countries given that clustering of health outcomes is a universal issue in human ageing that crosses national boundaries.

The limitations of this study should be noted. First, our analysis of childhood adversities is based on retrospective information. The recollection of childhood adversities or traumatic events may not be completely accurate. This recall bias is likely to affect older people aged over 80 more because those events took place in the distant past and some people might experience a memory decline. Indeed, recall bias may provide another possible explanation for the weakened association between childhood adversities and healthy ageing among people in the higher age groups. Second, due to the availability of data, we were not able to include genetic factors that confound the relationships between childhood adversities and healthy ageing, which to some extent limited our ability to interpret the association identified in the analysis as causality. Future analysis based on the prospective information will be valuable to mitigate these limitations. Finally, the k-means clustering technique is a data-driven method, so the grouping categories are sample specific. The thresholds of healthy ageing derived from one sample cannot be directly applied to another one.

## Conclusions

Healthy ageing is influenced by events and experiences throughout the life course, among which childhood adversities are significant risk factors. Policies and services that directly target older people, such as high-quality healthcare, long-term care, and an age-friendly environment, are imperative to healthy ageing, but the life course perspective suggests that policy responses should not be confined to these. We argue that the issue of healthy ageing can be better addressed by concerted efforts in different areas of social policy. Social programmes that provide training on good parenting practices not only ensure that children grow up in a supportive and protective environment, but also sow the seeds for healthy ageing that will materialise decades later. In a similar vein, gender equality policies play an irreplaceable role in upholding social justice and creating equal opportunities for women in the workplace and society. In this study, we want to stress that they are equally important in terms of building women’s resilience to the life course risks of health and reducing the gender gap in healthy ageing.

## Data Availability

The CHARLS dataset used in this study is publicly available (http://charls.pku.edu.cn/pages/data/2015-charls-wave4/en.html); Stata code used to analyse the data will be available upon request.

## Appendix

See Tables

**Table 5:** Questions used to construct the cognitive functioning variable (CHARLS 2015, *N* = 9248)

Cognitive functioning questions	Proportion of correct answer (%)
*Could you please tell me today’s date?*	
Year	65
Month	76
Day	58
Could you please tell me the day of the week?	56
What is the current season?	77
What does 100 minus 7 equal?	75
And 7 from that?	51
And 7 from that?	40
And 7 from that?	35
And 7 from that?	31
Filling in the missing number (7, 8,_,10)	71
Filling in the missing number 2 (8, _, 12, 14)	32
Filling in the missing number 3 (18, 10, 6, _, 3)	22
Do you see this picture? Please draw it on the paper	48
*How would you rate your memory at the present time?*	
Good or fair	61
Poor	39

^5^ and

**Table 6:** Proportion of missing values in the variables before imputation (CHARLS 2015, *N* = 9248)

Variables	Proportion of missing value (%)
ADL scores	0.3
IADL scores	0.3
Life satisfaction	7.4
Depression scores	16.6
Self-reported health	6.2
Number of chronic diseases	0.3
Cognitive functioning (excluding word recalls)	6.2
Bullying victimisation	5.0
Poor childhood health	1.2
Other childhood adversities	< 1
Age	0.5
Education	0.1
Marital status	0.02
Living arrangements	0.1
Housing tenure	9.5
Income	9.8
Word recalls—first round	> 46.0
Word recalls—second round	> 59.2

The variables relating to word recalls were excluded from the analyses due to a substantial proportion of missing values

6.
